# Synthesis, crystal structure and Hirshfeld surface analysis of sodium bis­(malonato)borate monohydrate

**DOI:** 10.1107/S2056989024000537

**Published:** 2024-01-26

**Authors:** Ramalingam Selvi, Govindharajan Gokila, Aravazhi Amalan Thiruvalluvar, Raju Sarangapani Sundararajan

**Affiliations:** a Department of Physics, Government College for Women (Autonomous), (affiliated to Bharathidasan University), Kumbakonam 612 001, Tamilnadu, India; b Principal (Retired), Kunthavai Naacchiyaar Government Arts College for Women (Autonomous), Thanjavur 613 007, Tamilnadu, India; c Department of Physics, Government Arts College (Autonomous), (affiliated to Bharathidasan University), Kumbakonam 612 002, Tamilnadu, India; University of Aberdeen, United Kingdom

**Keywords:** synthesis, crystal structure, bis­(malonato)borate anion, sodium, Hirshfeld surface analysis.

## Abstract

In the title salt, poly[aqua­[μ_4_-bis­(malonato)borato]sodium], {[Na(C_6_H_4_BO_8_)]·H_2_O}_*n*_ or Na^+^·[B(C_3_H_2_O_4_)_2_]^−^·H_2_O, the sodium cation exhibits fivefold coordination by four carbonyl O atoms of the bis­(malonato)borate anions and a water O atom. The tetra­hedral B atom at the centre of the anion leads to the formation of a polymeric three-dimensional framework, which is consolidated by C—H⋯O and O—H⋯O hydrogen bonds. A Hirshfeld surface analysis indicates that the most significant contacts in the crystal packing are H⋯O/O⋯H (49.7%), Na⋯O/O⋯Na (16.1%), O⋯O (12.6%), H⋯H (10.7%) and C⋯O/O⋯C (7.3%).

## Abstract 2

The asymmetric unit of the title salt, Na^+^·C_6_H_4_BO_8_^−^·H_2_O, com­prises a five-coordinate sodium cation, a bis­(malonato)borate anion and a water mol­ecule of crystallization.

## 1. Chemical context

The review by Vaalma *et al.* (2018 ▸) provides a com­prehensive overview of the cost and resource implications of sodium-ion batteries, which are a promising alternative to lithium-ion batteries for energy storage applications. The authors con­clude that sodium-ion batteries have the potential to be significantly less expensive than lithium-ion batteries, due to the abundance of sodium and the lower cost of sodium-based materials. Allen *et al.* (2012 ▸) described the structure of lithium bis­(2-methyl­lactato)borate monohydrate and there is a growing inter­est, as highlighted in various recent studies (Vaalma *et al.*, 2018 ▸; Abraham, 2020 ▸; Li *et al.*, 2019 ▸; Wang *et al.*, 2018 ▸), in lithium bis­(malonato)borate polymers as robust electrolytes. 
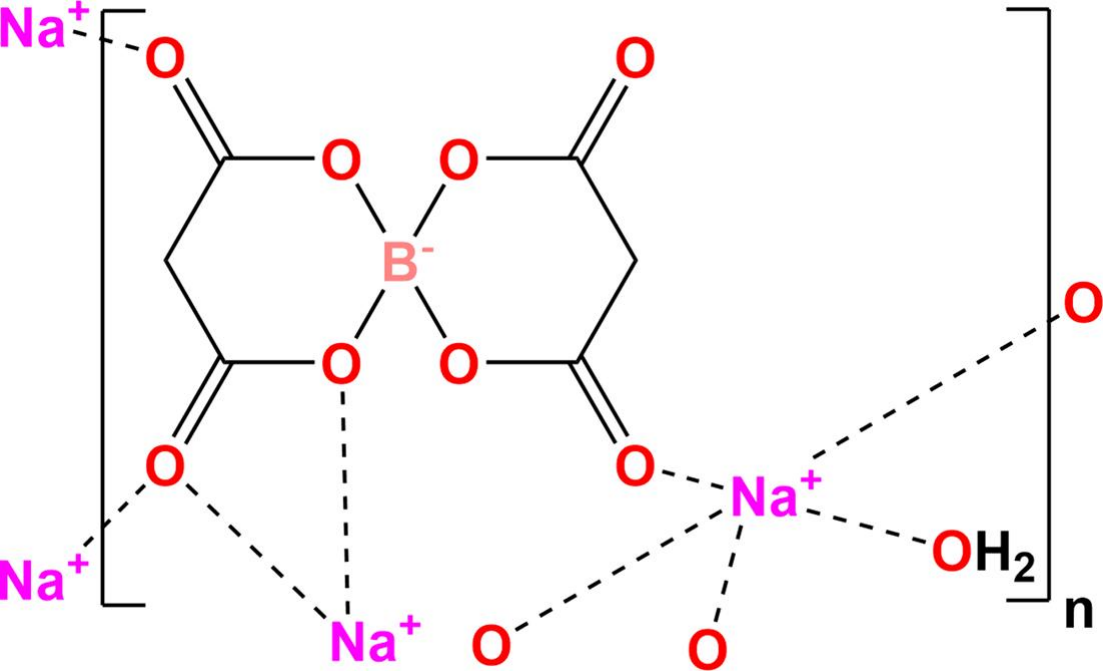


The present study explores the substitution of 2-methyl­lactic acid and lithium carbonate with malonic acid and sodium carbonate, respectively, and presents the synthesis, crystal structure and Hirshfeld surface analysis of the title com­pound, Na^+^·[B(C_3_H_2_O_4_)_2_]^−^·H_2_O, (I).

## 2. Structural commentary

**Figure 1:**
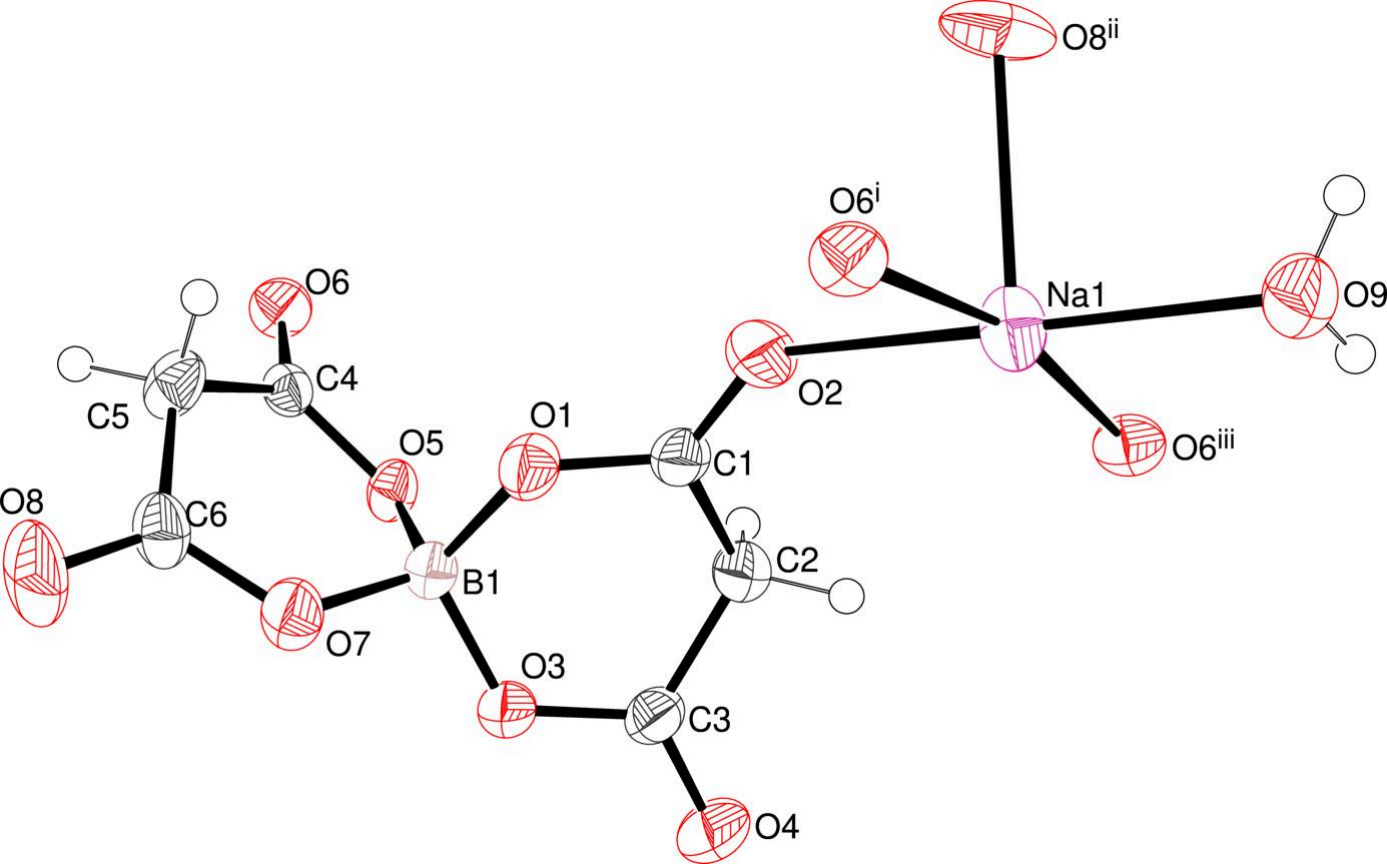
View of the mol­ecular structure of (I), showing 50% probability displacement ellipsoids (arbitrary spheres for the H atoms).

The asymmetric unit of (I) com­prises a sodium cation, a bis­(malonato)borate anion and a coordinated water mol­ecule (Fig. 1 ▸). The tetra­hedral B atom is bonded to two malonate (C_3_H_2_O_4_) ligands coordinated in an *O*,*O*′-bidentate mode.

In the boron–oxygen tetra­hedron, the mean B—O bond length of 1.4641 Å is in good agreement with the expected B(*sp* ^3^)—O bond length of 1.468 Å (Allen *et al.*, 1987 ▸). The largest O—B—O bond angles are the intra­cyclic angles: O1—B1—O3 = 112.92 (9)° and O5—B1—O7 = 112.21 (9)°. The six-membered boro–malonate rings O1/C1/C2/C3/O3/B1 and O5/C4/C5/C6/O7/B1 both adopt boat conformations [puckering parameters *Q* = 0.4082 (13) Å, θ = 87.85 (18)° and φ = 309.10 (18)° for the first ring, and *Q* = 0.4267 (13) Å, θ = 84.32 (16)° and φ = 317.00 (17)° for the second ring]. The ‘prow and stern’ atoms in the first ring, B1 and C2, are displaced by 0.3680 (18) and 0.330 (2) Å, respectively, from C1/C3/O1/O3 (r.m.s. deviation = 0.0323 Å). In the second ring, the equivalent data are a B1 displacement of 0.4048 (17), a C5 displacement of 0.298 (2) Å and an r.m.s. deviation of 0.0621 Å. The dihedral angle between the O1/C1/C3/O3 and O5/C4/C6/O7 least-squares planes is 73.34 (4)°.

Na1 is surrounded by five O atoms [carbonyl O2 and O6 at (*x* − 1, *y*, *z*), water O9, carbonyl O8 at (*x* − 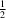 , −*y* + 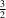 , *z* + 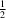 ) and O6 at (−*x* + 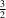 , *y* − 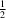 , −*z* + 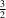 )], forming a square-based pyramidal coordination polyhedron (Table 1 ▸). A possible sixth bond [Na1—O5 at (*x* − 1, *y*, *z*)], which would generate a distorted octa­hedron, is much longer at 3.0195 (10) Å and presumably only represents a marginal inter­action in the structure.

## 3. Supra­molecular features and Hirshfeld surface analysis

**Figure 2:**
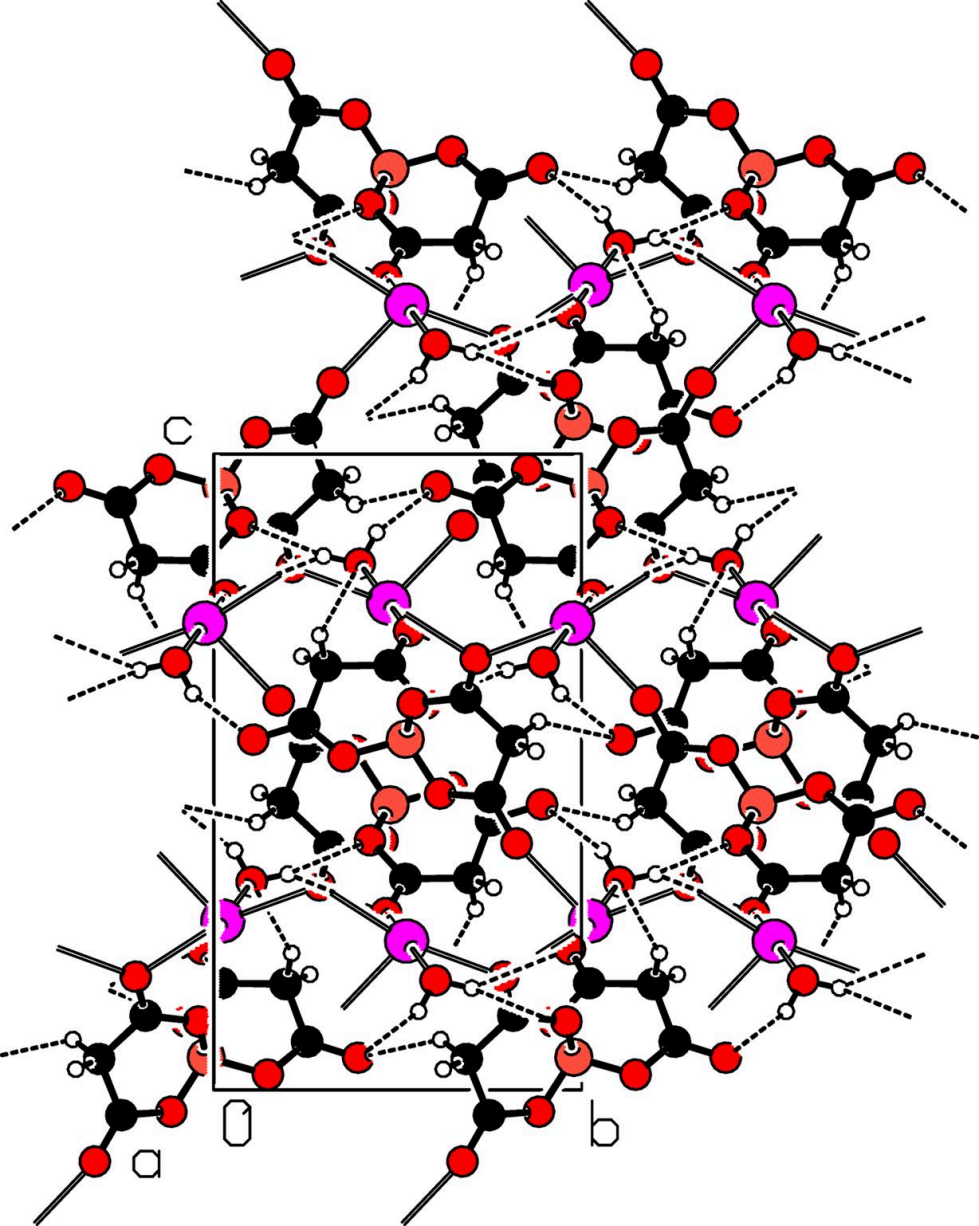
A packing diagram of (I), viewed along the *a*-axis direction (projection onto the *bc* plane), showing the C—H⋯O and O—H⋯O hydrogen bonds as black dashed lines.

The crystal structure of (I) is consolidated by two C—H⋯O links with the acceptors being one water O9 atom and one carbonyl O4 atom, and three O—H⋯O links, one of which is bifurcated, with the acceptors being one borate O1 atom and carbonyl atoms O4 and O2 (Table 2 ▸). The packing of the structure is shown in Fig. 2 ▸.

**Figure 3:**
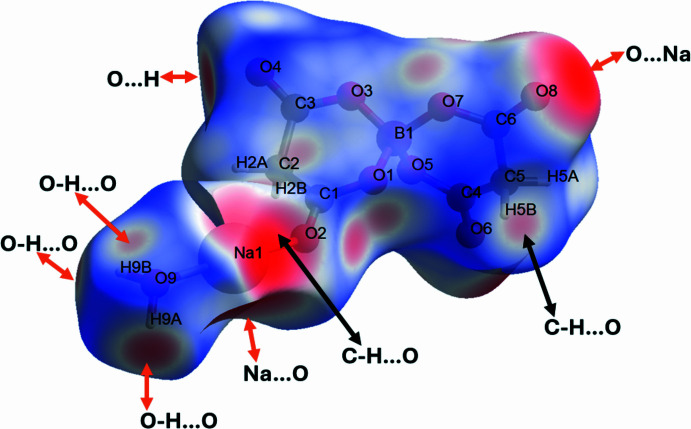
The Hirshfeld surface for (I). The surface is drawn with transparency and simplified for clarity, and the regions with the strongest inter­molecular inter­actions are shown in red. (*d*_norm_ range: −0.66 to +0.93 a.u.)

**Figure 4:**
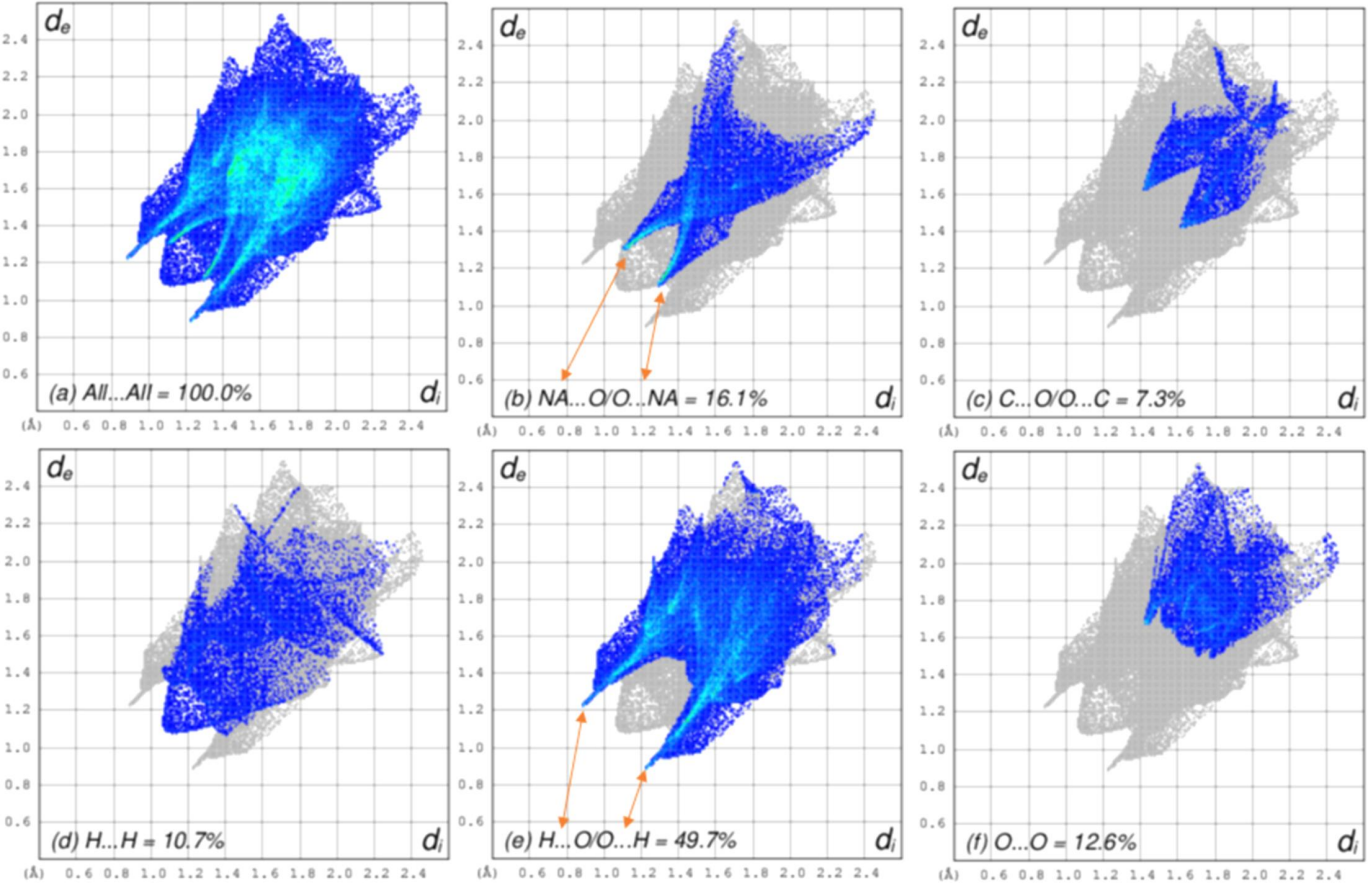
A view of the two-dimensional fingerprint plots for title com­pound (I), showing (*a*) all inter­actions, and those delineated into (*b*) Na⋯O/O⋯Na, (*c*) C⋯O/O⋯C, (*d*) H⋯H, (*e*) H⋯O/O⋯H and (*f*) O⋯O inter­actions. The *d*_i_ (*x* axis) and *d*_e_ (*y* axis) values are the closest inter­nal and external distances (in Å) from given points on the Hirshfeld surface.

The Hirshfeld surface analysis of (I) was performed with *CrystalExplorer* (Version 21.5; Spackman *et al.*, 2021 ▸). Fig. 3 ▸ shows the *d*_norm_ surface for the bis­(malonato)borate anion plotted over the limits from −0.66 to +0.93 a.u. The intense red spots represent the shortest inter­molecular contacts (attractive inter­actions like hydrogen bonds) and the blue regions denotes the longest (indicating possible repulsive inter­actions or regions with weak van der Waals inter­actions). Fig. 4 ▸ presents the two-dimensional fingerprint plots involving all the inter­molecular inter­actions [Fig. 4 ▸(*a*)] and delineated into Na⋯O/O⋯Na = 16.1% [Fig. 4 ▸(*b*)], C⋯O/O⋯C = 7.3% [Fig. 4 ▸(*c*)], H⋯H = 10.7% [Fig. 4 ▸(*d*)], H⋯O/O⋯H = 49.7% [Fig. 4 ▸(*e*)] and O⋯O = 12.6% [Fig. 4 ▸(*f*)] inter­actions. The remaining inter­actions contribute less than 2.0%. The hydrogen bonds are indicated by pairs of characteristic wings in the fingerprint plot [Fig. 4 ▸(*e*)] representing H⋯O/O⋯H contacts. Pairs of scattered points of spikes are seen in the fingerprint plot delineated into H⋯O/O⋯H contacts (49.7% the maximum contribution to the Hirshfeld surface) [Fig. 4 ▸(*e*)].

## 4. Database survey

A search using CCDC *ConQuest* of the Cambridge Structural Database (CSD, Version 5.44, updated to June 2023; Groom *et al.*, 2016 ▸) for the bis­(malonato)borate anion gave one hit (CSD refcode PITQUF; Zviedre & Belyakov, 2007 ▸), in which the bis­(malonato)borate unit is similar to that in (I) and is charge balanced by potassium cations. The K^+^ coordination geometry in PITQUF is an irregular nine-vertex polyhedron formed by the O atoms of seven com­plex anions.

## 5. Synthesis and crystallization

The title com­pound was synthesized by mixing malonic acid (C_3_H_4_O_4_), boric acid (H_3_BO_3_) and sodium carbonate (Na_2_CO_3_) in a 4:2:1 molar ratio, using deionized water as the solvent. Initially, malonic acid (4.1264 g) was dissolved in deionized water. This was followed by the addition of a boric acid solution (1.236 g) and then a sodium carbonate solution (1.382 g) was added. The mixture was stirred thoroughly to ensure a uniform solution. The beaker containing the solution was then covered with a perforated sheet and left undisturbed. Over three months, due to slow evaporation of the solvent, small clear crystals of (I) formed at the bottom of the container [yield: 2.744 g, 40.6%; m.p. 527–529 K (decom­position)]. FT–IR (cm^−1^): 3928, 3856, 3419, 3014, 2908, 2542, 2016, 1711, 1635, 1400, 1334, 1271, 1069, 1019, 958, 914, 859, 833, 657, 634, 561, 478, 457, 423.

## 6. Refinement

Crystal data and structure refinement details are summarized in Table 3 ▸. The H atoms attached to C atoms were placed in calculated positions (C—H = 0.97 Å) and refined as riding atoms with *U*_iso_(H) = 1.2*U*_eq_(C). The water H atoms were located in difference maps and refined with restraints (O—H = 0.84 ± 0.02 Å and H⋯H = 1.36 ± 0.02 Å) to ensure a realistic geometry.

## Supplementary Material

Crystal structure: contains datablock(s) global. DOI: 10.1107/S2056989024000537/hb8086sup1.cif

CCDC reference: 2325391

Additional supporting information:  crystallographic information; 3D view; checkCIF report

## Figures and Tables

**Table 1:** Selected geometric parameters (Å, °)

Na1—O9	2.3264 (12)	B1—O3	1.4508 (15)
Na1—O2	2.3402 (11)	B1—O7	1.4581 (16)
Na1—O6^i^	2.4032 (10)	B1—O1	1.4697 (15)
Na1—O8^ii^	2.4213 (12)	B1—O5	1.4779 (15)
Na1—O6^iii^	2.4455 (11)		
			
O9—Na1—O2	171.38 (5)	O6^i^—Na1—O8^ii^	80.15 (4)
O9—Na1—O6^i^	102.48 (4)	O9—Na1—O6^iii^	84.03 (4)
O2—Na1—O6^i^	85.61 (4)	O2—Na1—O6^iii^	88.60 (4)
O9—Na1—O8^ii^	88.27 (5)	O6^i^—Na1—O6^iii^	167.40 (3)
O2—Na1—O8^ii^	90.22 (5)	O8^ii^—Na1—O6^iii^	111.10 (5)

Symmetry codes: (i) 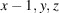 ; (ii) 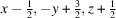 ; (iii) 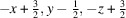 .

**Table 2:** Hydrogen-bond geometry (Å, °)

*D*—H⋯*A*	*D*—H	H⋯*A*	*D*⋯*A*	*D*—H⋯*A*
C2—H2*B*⋯O9^iv^	0.97	2.54	3.4247 (18)	151
C5—H5*B*⋯O4^v^	0.97	2.46	3.2830 (18)	143
O9—H9*A*⋯O4^vi^	0.85 (1)	2.23 (2)	2.9932 (17)	151 (3)
O9—H9*B*⋯O1^vii^	0.84 (1)	2.46 (2)	3.1520 (14)	140 (3)
O9—H9*B*⋯O2^vii^	0.84 (1)	2.39 (2)	3.1166 (17)	146 (3)

Symmetry codes: (iv) 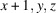 ; (v) 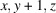 ; (vi) 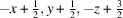 ; (vii) 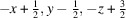 .

**Table 3:** Experimental details

Crystal data	
Chemical formula	[Na(C_6_H_4_BO_8_)(H_2_O)]
*M*_r_	255.91
Crystal system, space group	Monoclinic, *P*2_1_/*n*
Temperature (K)	298
*a*, *b*, *c* (Å)	7.9058 (4), 8.2979 (5), 14.6473 (9)
β (°)	101.565 (2)
*V* (Å^3^)	941.38 (9)
*Z*	4
Radiation type	Mo *K*α
μ (mm^−1^)	0.21
Crystal size (mm)	0.35 × 0.27 × 0.27

Data collection	
Diffractometer	Bruker D8 VENTURE diffrac­tometer with a PHOTON II detector
Absorption correction	Multi-scan (*SADABS*; Krause *et al.*, 2015 ▸)
*T*_min_, *T*_max_	0.917, 0.958
No. of measured, independent and observed [*I* > 2σ(*I*)] reflections	32209, 2864, 2494
*R*_int_	0.030
(sin θ/λ)_max_ (Å^−1^)	0.714

Refinement	
*R*[*F* ^2^ > 2σ(*F* ^2^)], *wR*(*F* ^2^), *S*	0.038, 0.105, 1.08
No. of reflections	2864
No. of parameters	162
No. of restraints	4
H-atom treatment	H atoms treated by a mixture of independent and constrained refinement
Δρ_max_, Δρ_min_ (e Å^−3^)	0.53, −0.60

Computer programs: *APEX4*, *SAINT* and *XPREP* (Bruker, 2021 ▸), *SHELXT* (Sheldrick, 2015*a* ▸), *SHELXL* (Sheldrick, 2015*b* ▸), *ORTEP-3 for Windows* (Farrugia, 2012 ▸), *PLATON* (Spek, 2020 ▸) and *publCIF* (Westrip, 2010 ▸).

## supplementary crystallographic information

### Crystal data

**Table:**

[Na(C_6_H_4_BO_8_)(H_2_O)]	*F*(000) = 520
*M**_r_* = 255.91	*D*_x_ = 1.806 Mg m^−^^3^
Monoclinic, *P*2_1_/*n*	Mo *K*α radiation, λ = 0.71073 Å
*a* = 7.9058 (4) Å	Cell parameters from 9951 reflections
*b* = 8.2979 (5) Å	θ = 2.8–30.5°
*c* = 14.6473 (9) Å	µ = 0.21 mm^−^^1^
β = 101.565 (2)°	*T* = 298 K
*V* = 941.38 (9) Å^3^	Block, colourless
*Z* = 4	0.35 × 0.27 × 0.27 mm

### Data collection

**Table:**

Bruker D8 VENTURE diffractometer with a PHOTON II detector	2494 reflections with *I* > 2σ(*I*)
Radiation source: fine-focus sealed tube	*R*_int_ = 0.030
ω and φ scan	θ_max_ = 30.5°, θ_min_ = 3.6°
Absorption correction: multi-scan (*SADABS*; Krause *et al.*, 2015)	*h* = −10→11
*T*_min_ = 0.917, *T*_max_ = 0.958	*k* = −11→11
32209 measured reflections	*l* = −20→20
2864 independent reflections	

### Refinement

**Table:**

Refinement on *F*^2^	Primary atom site location: dual
Least-squares matrix: full	Secondary atom site location: difference Fourier map
*R*[*F*^2^ > 2σ(*F*^2^)] = 0.038	Hydrogen site location: mixed
*wR*(*F*^2^) = 0.105	H atoms treated by a mixture of independent and constrained refinement
*S* = 1.08	*w* = 1/[σ^2^(*F*_o_^2^) + (0.0503*P*)^2^ + 0.3262*P*] where *P* = (*F*_o_^2^ + 2*F*_c_^2^)/3
2864 reflections	(Δ/σ)_max_ < 0.001
162 parameters	Δρ_max_ = 0.53 e Å^−^^3^
4 restraints	Δρ_min_ = −0.60 e Å^−^^3^

### Special details

**Table:**

Geometry. All esds (except the esd in the dihedral angle between two l.s. planes) are estimated using the full covariance matrix. The cell esds are taken into account individually in the estimation of esds in distances, angles and torsion angles; correlations between esds in cell parameters are only used when they are defined by crystal symmetry. An approximate (isotropic) treatment of cell esds is used for estimating esds involving l.s. planes.

### Fractional atomic coordinates and isotropic or equivalent isotropic displacement parameters (Å^2^)

**Table:**

	*x*	*y*	*z*	*U*_iso_*/*U*_eq_	
Na1	0.24488 (7)	0.47538 (7)	0.76584 (4)	0.03728 (15)	
B1	0.76757 (17)	0.52073 (15)	0.55156 (9)	0.0271 (2)	
C1	0.59169 (15)	0.47853 (15)	0.66789 (9)	0.0294 (2)	
C2	0.64385 (17)	0.30481 (15)	0.66658 (9)	0.0331 (3)	
H2A	0.551966	0.239128	0.681940	0.040*	
H2B	0.745590	0.288268	0.715054	0.040*	
C3	0.68228 (16)	0.24605 (14)	0.57587 (9)	0.0309 (2)	
C4	1.01449 (13)	0.68524 (13)	0.61701 (7)	0.0235 (2)	
C5	0.93606 (17)	0.81205 (14)	0.54883 (9)	0.0340 (3)	
H5A	1.028548	0.875823	0.532601	0.041*	
H5B	0.867448	0.883229	0.579401	0.041*	
C6	0.82428 (16)	0.75128 (16)	0.46059 (9)	0.0331 (3)	
O1	0.64336 (12)	0.57513 (11)	0.60743 (7)	0.0337 (2)	
O2	0.50736 (14)	0.53111 (13)	0.72169 (8)	0.0451 (3)	
O3	0.74511 (13)	0.35276 (11)	0.52435 (7)	0.0358 (2)	
O4	0.66050 (17)	0.10738 (13)	0.55087 (9)	0.0518 (3)	
O5	0.94362 (11)	0.54098 (10)	0.60778 (6)	0.02768 (18)	
O6	1.13921 (12)	0.71217 (11)	0.67822 (6)	0.0329 (2)	
O7	0.74022 (12)	0.61563 (12)	0.46608 (6)	0.0366 (2)	
O8	0.80823 (17)	0.82359 (17)	0.38773 (8)	0.0577 (3)	
O9	0.00848 (15)	0.39690 (13)	0.82757 (8)	0.0448 (3)	
H9A	−0.008 (4)	0.443 (3)	0.8765 (14)	0.088 (9)*	
H9B	0.019 (4)	0.2983 (16)	0.8397 (19)	0.100 (10)*	

### Atomic displacement parameters (Å^2^)

**Table:**

	*U*^11^	*U*^22^	*U*^33^	*U*^12^	*U*^13^	*U*^23^
Na1	0.0334 (3)	0.0373 (3)	0.0430 (3)	0.0070 (2)	0.0124 (2)	0.0112 (2)
B1	0.0289 (6)	0.0257 (6)	0.0283 (6)	−0.0034 (4)	0.0090 (5)	−0.0002 (4)
C1	0.0242 (5)	0.0316 (6)	0.0337 (6)	−0.0052 (4)	0.0089 (4)	−0.0065 (4)
C2	0.0401 (6)	0.0291 (6)	0.0316 (6)	−0.0021 (5)	0.0107 (5)	0.0008 (4)
C3	0.0322 (5)	0.0252 (5)	0.0360 (6)	−0.0037 (4)	0.0086 (4)	−0.0039 (4)
C4	0.0255 (4)	0.0238 (5)	0.0219 (4)	0.0022 (4)	0.0062 (4)	0.0025 (4)
C5	0.0391 (6)	0.0234 (5)	0.0355 (6)	−0.0004 (4)	−0.0022 (5)	0.0087 (5)
C6	0.0310 (5)	0.0382 (6)	0.0286 (6)	0.0004 (5)	0.0025 (4)	0.0120 (5)
O1	0.0349 (4)	0.0249 (4)	0.0456 (5)	0.0004 (3)	0.0180 (4)	−0.0013 (4)
O2	0.0423 (5)	0.0478 (6)	0.0527 (6)	−0.0066 (4)	0.0272 (5)	−0.0150 (5)
O3	0.0458 (5)	0.0288 (4)	0.0375 (5)	−0.0077 (4)	0.0198 (4)	−0.0079 (4)
O4	0.0722 (8)	0.0270 (5)	0.0611 (7)	−0.0110 (5)	0.0249 (6)	−0.0122 (5)
O5	0.0295 (4)	0.0236 (4)	0.0292 (4)	−0.0007 (3)	0.0042 (3)	0.0075 (3)
O6	0.0332 (4)	0.0349 (4)	0.0274 (4)	−0.0009 (3)	−0.0016 (3)	0.0013 (3)
O7	0.0369 (4)	0.0411 (5)	0.0282 (4)	−0.0087 (4)	−0.0017 (3)	0.0066 (4)
O8	0.0601 (7)	0.0713 (8)	0.0370 (6)	−0.0081 (6)	−0.0020 (5)	0.0315 (6)
O9	0.0525 (6)	0.0366 (5)	0.0487 (6)	−0.0035 (4)	0.0181 (5)	0.0039 (5)

### Geometric parameters (Å, º)

**Table:**

Na1—O9	2.3264 (12)	C2—H2A	0.9700
Na1—O2	2.3402 (11)	C2—H2B	0.9700
Na1—O6^i^	2.4032 (10)	C3—O4	1.2094 (16)
Na1—O8^ii^	2.4213 (12)	C3—O3	1.3231 (15)
Na1—O6^iii^	2.4455 (11)	C4—O6	1.2130 (14)
Na1—O5^i^	3.0195 (10)	C4—O5	1.3171 (13)
B1—O3	1.4508 (15)	C4—C5	1.4969 (15)
B1—O7	1.4581 (16)	C5—C6	1.4993 (18)
B1—O1	1.4697 (15)	C5—H5A	0.9700
B1—O5	1.4779 (15)	C5—H5B	0.9700
C1—O2	1.2109 (15)	C6—O8	1.2086 (15)
C1—O1	1.3186 (15)	C6—O7	1.3177 (16)
C1—C2	1.5005 (17)	O9—H9A	0.846 (13)
C2—C3	1.5024 (17)	O9—H9B	0.838 (13)
			
O9—Na1—O2	171.38 (5)	O4—C3—C2	122.33 (12)
O9—Na1—O6^i^	102.48 (4)	O3—C3—C2	116.93 (10)
O2—Na1—O6^i^	85.61 (4)	O6—C4—O5	120.62 (10)
O9—Na1—O8^ii^	88.27 (5)	O6—C4—C5	122.02 (10)
O2—Na1—O8^ii^	90.22 (5)	O5—C4—C5	117.36 (10)
O6^i^—Na1—O8^ii^	80.15 (4)	O6—C4—Na1^iv^	45.91 (6)
O9—Na1—O6^iii^	84.03 (4)	O5—C4—Na1^iv^	74.82 (6)
O2—Na1—O6^iii^	88.60 (4)	C5—C4—Na1^iv^	167.29 (8)
O6^i^—Na1—O6^iii^	167.40 (3)	C4—C5—C6	115.62 (10)
O8^ii^—Na1—O6^iii^	111.10 (5)	C4—C5—H5A	108.4
O9—Na1—O5^i^	77.10 (4)	C6—C5—H5A	108.4
O2—Na1—O5^i^	111.06 (4)	C4—C5—H5B	108.4
O6^i^—Na1—O5^i^	46.12 (3)	C6—C5—H5B	108.4
O8^ii^—Na1—O5^i^	117.12 (4)	H5A—C5—H5B	107.4
O6^iii^—Na1—O5^i^	127.08 (4)	O8—C6—O7	120.86 (13)
O3—B1—O7	107.10 (10)	O8—C6—C5	122.25 (13)
O3—B1—O1	112.92 (9)	O7—C6—C5	116.88 (10)
O7—B1—O1	108.16 (10)	C1—O1—B1	121.25 (10)
O3—B1—O5	108.18 (10)	C1—O2—Na1	137.99 (9)
O7—B1—O5	112.21 (9)	C3—O3—B1	121.70 (10)
O1—B1—O5	108.33 (10)	C4—O5—B1	119.62 (9)
O2—C1—O1	120.21 (12)	C4—O5—Na1^iv^	80.28 (6)
O2—C1—C2	122.89 (12)	B1—O5—Na1^iv^	156.45 (7)
O1—C1—C2	116.90 (10)	C4—O6—Na1^iv^	112.84 (8)
C1—C2—C3	115.33 (11)	C4—O6—Na1^v^	127.34 (8)
C1—C2—H2A	108.4	Na1^iv^—O6—Na1^v^	118.98 (3)
C3—C2—H2A	108.4	C6—O7—B1	121.60 (10)
C1—C2—H2B	108.4	C6—O8—Na1^vi^	164.30 (13)
C3—C2—H2B	108.4	Na1—O9—H9A	118 (2)
H2A—C2—H2B	107.5	Na1—O9—H9B	108 (2)
O4—C3—O3	120.73 (12)	H9A—O9—H9B	107 (2)
			
O2—C1—C2—C3	156.38 (13)	C5—C4—O5—B1	−17.36 (15)
O1—C1—C2—C3	−24.22 (16)	Na1^iv^—C4—O5—B1	166.54 (9)
C1—C2—C3—O4	−150.74 (13)	O6—C4—O5—Na1^iv^	−3.33 (10)
C1—C2—C3—O3	30.58 (17)	C5—C4—O5—Na1^iv^	176.10 (10)
O6—C4—C5—C6	160.61 (11)	O3—B1—O5—C4	159.43 (9)
O5—C4—C5—C6	−18.81 (16)	O7—B1—O5—C4	41.49 (14)
Na1^iv^—C4—C5—C6	143.8 (3)	O1—B1—O5—C4	−77.85 (12)
C4—C5—C6—O8	−150.46 (14)	O3—B1—O5—Na1^iv^	−55.6 (2)
C4—C5—C6—O7	30.90 (17)	O7—B1—O5—Na1^iv^	−173.55 (12)
O2—C1—O1—B1	170.39 (12)	O1—B1—O5—Na1^iv^	67.1 (2)
C2—C1—O1—B1	−9.02 (16)	O5—C4—O6—Na1^iv^	4.47 (13)
O3—B1—O1—C1	35.76 (16)	C5—C4—O6—Na1^iv^	−174.93 (9)
O7—B1—O1—C1	154.11 (10)	O5—C4—O6—Na1^v^	−164.81 (7)
O5—B1—O1—C1	−84.03 (13)	C5—C4—O6—Na1^v^	15.79 (16)
O1—C1—O2—Na1	126.39 (13)	Na1^iv^—C4—O6—Na1^v^	−169.29 (12)
C2—C1—O2—Na1	−54.2 (2)	O8—C6—O7—B1	175.44 (14)
O4—C3—O3—B1	178.15 (13)	C5—C6—O7—B1	−5.90 (18)
C2—C3—O3—B1	−3.14 (18)	O3—B1—O7—C6	−147.33 (11)
O7—B1—O3—C3	−147.88 (11)	O1—B1—O7—C6	90.69 (14)
O1—B1—O3—C3	−28.92 (17)	O5—B1—O7—C6	−28.75 (16)
O5—B1—O3—C3	90.96 (13)	O7—C6—O8—Na1^vi^	117.5 (4)
O6—C4—O5—B1	163.22 (10)	C5—C6—O8—Na1^vi^	−61.0 (5)

Symmetry codes: (i) *x*−1, *y*, *z*; (ii) *x*−1/2, −*y*+3/2, *z*+1/2; (iii) −*x*+3/2, *y*−1/2, −*z*+3/2; (iv) *x*+1, *y*, *z*; (v) −*x*+3/2, *y*+1/2, −*z*+3/2; (vi) *x*+1/2, −*y*+3/2, *z*−1/2.

### Hydrogen-bond geometry (Å, º)

**Table:**

*D*—H···*A*	*D*—H	H···*A*	*D*···*A*	*D*—H···*A*
C2—H2*B*···O9^iv^	0.97	2.54	3.4247 (18)	151
C5—H5*B*···O4^vii^	0.97	2.46	3.2830 (18)	143
O9—H9*A*···O4^viii^	0.85 (1)	2.23 (2)	2.9932 (17)	151 (3)
O9—H9*B*···O1^ix^	0.84 (1)	2.46 (2)	3.1520 (14)	140 (3)
O9—H9*B*···O2^ix^	0.84 (1)	2.39 (2)	3.1166 (17)	146 (3)

Symmetry codes: (iv) *x*+1, *y*, *z*; (vii) *x*, *y*+1, *z*; (viii) −*x*+1/2, *y*+1/2, −*z*+3/2; (ix) −*x*+1/2, *y*−1/2, −*z*+3/2.
